# Fuzheng Jiedu granules against disease progression among high-risk adults with non-severe COVID-19: a multicenter retrospective cohort study

**DOI:** 10.3389/fphar.2025.1523004

**Published:** 2025-05-12

**Authors:** Qiaoli Hua, Danwen Zheng, Jingwei Shui, Tong Zhang, Shengle Qin, Hanhong Zhang, Bo Yu, Longde Wang, Hailang He, Xinghua Tan, Qiumin Chen, Yang Yang, Weng Heng, Yihang Cai, Xiaohua Xu, Qing Liu, Yuntao Liu, Rongyuan Yang, Zhongde Zhang

**Affiliations:** ^1^ Department of Clinical Laboratory, Shenzhen Traditional Chinese Medicine Hospital, The Fourth Clinical Medical College of Guangzhou University of Chinese Medicine, Shenzhen, China; ^2^ Department of Emergency, The Second Affiliated Hospital of Guangzhou University of Chinese Medicine, Guangzhou, China; ^3^ The Second Clinical College, Guangzhou University of Chinese Medicine, Guangzhou, China; ^4^ Department of emergency, Guangdong Provincial Key Laboratory of Research on Emergency in TCM, Guangzhou, China; ^5^ Department of Cardiology, The Second Affiliated Hospital of Guangzhou University of Chinese Medicine, Guangzhou, China; ^6^ The Zhen’s Miscellaneous Diseases School in Lingnan (Lingnan Zhenshi Miscellaneous Diseases Genre), The Second Affiliated Hospital of Guangzhou University of Chinese Medicine, Guangzhou, China; ^7^ Department of Emergency, Hainan Provincial Hospital of Traditional Chinese Medicine, Haikou, China; ^8^ Department of General Surgery, The No.2 People’s Hospital of Lanzhou, Lanzhou, China; ^9^ Affiliated Hospital of Gansu University of Traditional Chinese Medicine, Lanzhou, China; ^10^ Department of Respiratory and Critical Care Medicine, Jiangsu Provincial Hospital of Traditional Chinese Medicine, Nanjing, China; ^11^ Department of Traditional Chinese Medicine, Guangzhou Eighth People’s Hospital, Guangzhou Medical University, Guangzhou, China; ^12^ Department of Traditional Chinese Medicine, The First Affiliated Hospital of Xiamen University, Xiamen, China; ^13^ Department of emergency,Dalian Hospital of Traditional Chinese Medicine, Dalian, China; ^14^ State Key Laboratory of Dampness Syndrome of Chinese Medicine, The Second Affiliated Hospital of Guangzhou University of Chinese Medicine, Guangzhou, China; ^15^ The Second Affiliated Hospital of Guangzhou University of Chinese Medicine-Zhuhai Hospital, Zhuhai, China

**Keywords:** COVID-19, Fuzheng Jiedu granules, traditional Chinese medicine, high-risk patients, disease progression

## Abstract

**Background:**

Fuzheng Jiedu (FZJD) granules are widely used to treat coronavirus disease (COVID-19) since their market approval, but their clinical effectiveness remains uncertain. In this study, we aimed to evaluate the effectiveness of FZJD in reducing disease progression in high-risk adults with COVID-19.

**Methods:**

A multicenter, retrospective cohort study involving high-risk adults with non-severe COVID-19 was conducted in China from May 2021 to December 2022. The study was registered at the Chinese Clinical Trial Registry (ChiCTR2200058097; https://www.chictr.org.cn/bin/project/edit?pid=160010). Patients were categorized into two groups based on the administration of FZJD granules. The outcomes included disease progression, mechanical ventilation, intensive care unit (ICU) admission, and mortality. Propensity-score analyses and multivariable regression were performed to assess the effectiveness of FZJD granules. The effectiveness was further analyzed in different subgroups.

**Results:**

A total of 1,644 patients (54.7% female patients; mean age, 62.3 years) were included, with 27.4% (451/1,644) receiving FZJD granules. After propensity score matching (PSM), 320 FZJD granule receivers and 320 non-receivers were matched. Those receiving FZJD granules were associated with lower risks of disease progression [adjusted odds ratio (OR), 0.21; 95% confidence interval (CI), 0.06–0.73], mechanical ventilation (OR, 0.15; 95% CI, 0.03–0.66), and ICU admission (OR, 0.08; 95% CI, 0.01–0.64) than those not receiving FZJD granules. The lower risk of disease progression in the FZJD group was confirmed by multivariable regression analysis and various propensity-score analyses. Furthermore, subgroup analyses demonstrated significant treatment benefits in patients with moderate COVID-19 at admission (no progression to severe disease) or in those who were not fully vaccinated (OR, 0.06; 95% CI, 0.01–0.50).

**Conclusion:**

FZJD administration was significantly associated with a reduced risk of disease progression in high-risk adults with mild-to-moderate COVID-19.

## Graphical Abstract

**Figure:**
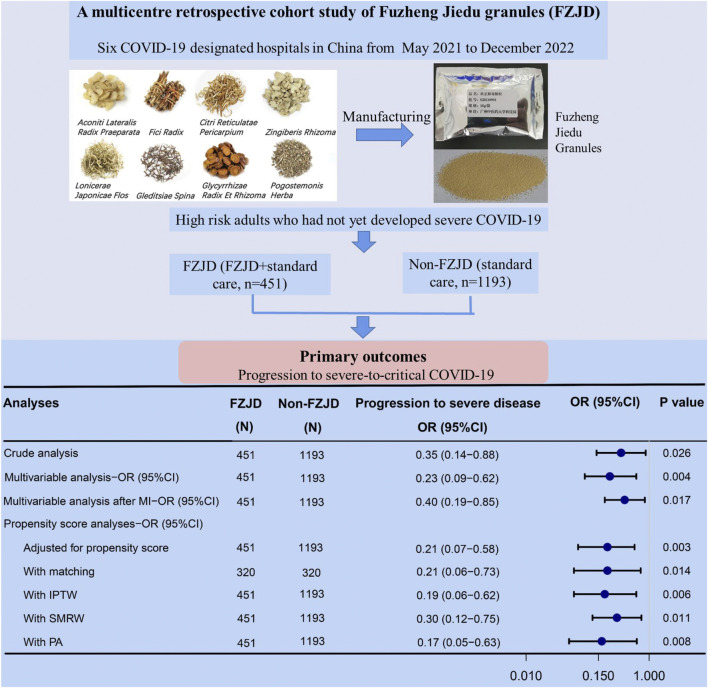

## 1 Introduction

The COVID-19 pandemic has been attenuated in most countries, attributed to viral evolution and global vaccination campaigns. However, progression to severe/critical disease continues to pose a significant medical challenge, especially for high-risk populations such as older adults and those with comorbidities (e.g., cardiovascular disease, diabetes, and chronic lung disease) (Ko et al., 2021; Zheng et al., 2020). Although vaccines are effective in reducing the incidence of severe disease in the general population, studies indicated that elderly adults may exhibit lower vaccine efficacy against severe acute respiratory syndrome coronavirus 2 (SARS-CoV-2) and its variants (Huang et al., 2024). To address this issue, early antiviral interventions such as Paxlovid (Hammond et al., 2022), monoclonal antibodies (Dougan et al., 2021), remdesivir (Gottlieb et al., 2022), and molnupiravir (Bernal et al., 2021) are potential options to prevent disease progression in these vulnerable individuals. However, these treatments remain underutilized due to accessibility and safety limitations. Thus, there is an urgent need for adjunctive therapies to prevent disease deterioration in non-severe but high-risk COVID-19 cases.

In China, from 2020 to 2022, more than 85% of patients with COVID-19 received Chinese herbal medicine (CHM) (Yang et al., 2020). CHM has been associated with several benefits, including improved clinical outcomes (Huang et al., 2021), faster recovery times (Shi et al., 2020), reduced mortality (Shu et al., 2021; Wang et al., 2021), and a lower incidence of severe disease (Tian et al., 2020). Fuzheng Jiedu (FZJD) granules, a traditional CHM formula, have been extensively employed in the management of COVID-19 patients, particularly those presenting with the syndrome of “Yang Qi Deficiency and Lingering Dampness-Toxin,” which is predominantly observed in high-risk patients. This formula originated from the classical CHM formula Sini Decoction (from *the Treatise on Febrile Diseases*). The core components of FZJD, Danfupian (*Aconiti Lateralis Radix Praeparata*), Ganjiang (*Zingiberis Rhizoma*), and Zhigancao (*Glycyrrhizae Radix Et Rhizoma*), work together to tonify Qi and warm Yang; Wuzhimaotao (*Fici Radix*) has the function of tonifying middle Qi; Jinyinhua (*Lonicerae Japonicae Flos*) and Zaojiaoci (*Gleditsiae Spina*) clear away heat and toxicity; and Chenpi (*Citri Reticulatae Pericarpium*) and Guanghuoxiang (*Pogostemonis Herba*) eliminate dampness and resolve phlegm. This herbal combination enhances the body’s resistance, detoxifies, and eliminates dampness. It has been recommended by *China’s COVID-19 Diagnosis and Treatment Guidelines* (*10th edition*) (National Health Comission and National Administration of Traditional Chinese Medicine, 2023a) and was approved for marketing in China by the National Medical Products Administration of the People’s Republic of China (renamed as Wenyang Jiedu Granules, Approval Number, Guoyao Zhunzi C20240004) (National Medical Products Administration, 2025).

To address the pharmacokinetic profile of FZJD, our preliminary study identified 203 constituents using ultra-high-performance liquid chromatography coupled with quadrupole time-of-flight mass spectrometry (UHPLC-Q-TOF/MS). This analysis revealed a diverse array of compounds, including 80 flavonoids, 24 triterpenoids, 16 iridoids, 39 organic acids, and 44 other metabolites. Following this, we conducted *in vivo* studies in both C57BL/6 and hACE2 transgenic mice, which demonstrated systemic exposure to 12 bioactive constituents after oral administration (40 g/kg). Among these bioactive constituents, liquiritin, hesperidin, liquiritigenin, isoliquiritigenin, and formononetin were consistently detected in the plasma, liver, lung, and urine in both models. Our pharmacokinetic analysis indicated that liquiritin, isoliquiritin, liquiritigenin, and isoliquiritigenin exhibited prolonged therapeutic windows, indicating their potential as core active constituents. Furthermore, these compounds have been proven to exhibit anti-inflammatory properties, thereby mitigating acute lung injury (Zhou et al., 2023; Yang et al., 2025; Hosawi, 2023; Chen et al., 2021) by modulating inflammatory pathways.

Further pharmacological research has indicated that FZJD exerted protective effects against acute lung injury induced by lipopolysaccharide (Lu et al., 2022) and the H1N1 virus (Li et al., 2025) in mice. This protection was evidenced by a significant reduction in the lung index, alleviation of histopathological injury, and suppression of inflammatory cytokines such as IL-1β, IL-6, and TNF-α (Lu et al., 2022; Li et al., 2025; Lu et al., 2024). Additionally, an *in vitro* study showed that FZJD-containing serum enhanced the integrity of the alveolar epithelial barrier (Chen et al., 2023). These beneficial effects were attributed to the regulation of multiple pathways, which included the inhibition of NLRP3 inflammasome activation (Li et al., 2025), amino acid metabolism modulation *via* gut–lung axis interactions (Lu et al., 2024), and the inhibition of PI3K/Akt signal pathway activation (Chen et al., 2023).

Although FZJD has shown anti-inflammatory and immunoregulatory properties (Lu et al., 2022; Li et al., 2025; Chen et al., 2023; Lu et al., 2024), limited clinical evidence exists regarding its clinical effectiveness (Wang et al., 2021). A previous retrospective study reported that FZJD may shorten the duration of fever and reduce the 28-day mortality rate in patients with COVID-19 (Wang et al., 2021). However, the small sample size in the study limited the strength of the evidence. Given the wide application of FZJD since its market approval, there is an urgent need for a comprehensive assessment of its effectiveness. Using the COVID-19 database from early 2021–2022, we conducted a multicenter retrospective cohort study aimed at investigating the effectiveness of a treatment regimen that combines FZJD with standard care, compared to standard care alone, in hospitalized high-risk COVID-19 patients.

## 2 Materials and methods

### 2.1 Study design

From 2021 to 2022, due to the “dynamic zero-COVID” policy, there were only sporadic local outbreaks of COVID-19 in China. According to this policy, all confirmed COVID-19 patients were required to be admitted to designated hospitals for COVID-19, regardless of disease severity. This retrospective cohort study was conducted during this phase between May 2021 and September 2022 in six designated COVID-19 hospitals in China, thus comprising a study population of 6,183 consecutive individuals. Additional information on these hospitals is provided in Supplementary Table S1.

This study was approved by the Ethical Committee of Guangdong Provincial Hospital of Chinese Medicine (No. BE2022-256-01) and registered at the Chinese Clinical Trial Registry (ChiCTR2200058097). The committee waived informed consent because of the study’s observational nature.

### 2.2 Study population and data sources

We analyzed the electronic medical records of patients with confirmed SARS-CoV-2 infection (defined by laboratory-confirmed positive RT-PCR tests) from the six designated hospitals. Data on demographic information, COVID-19 severity upon admission, vaccination status, date of symptom onset, epidemic waves and SARS-CoV-2 variants, comorbidities (e.g., hypertension, diabetes, and heart disease), concomitant therapies (e.g., respiratory support and antiviral agents), and clinical outcomes were obtained from hospital admission to discharge.

Adult patients (≥18 years) at high risk for severe COVID-19 who had not yet developed severe COVID-19 were included in the study. According to the *Diagnosis and Treatment Guidelines* [8th (National Health Comission and National Administration of Traditional Chinese Medicine, 2023b) and 9th (National Health Comission and National Administration of Traditional Chinese Medicine, 2023c)] in China, high-risk cases in this study referred to patients with at least one of the following characteristics: age ≥60 years, obesity [body mass index ≥30 kg/m^2^], heavy smoker, immune-compromised status (e.g., patients who were post-transplantation, receiving immune suppressive medications, or who were HIV-infected), and comorbidities (e.g., hypertension, chronic lung disease, diabetes, cancer, chronic liver disease, chronic kidney disease, or cardio-cerebrovascular disease).

Our study excluded patients who met the following criteria: ≥10 days from symptom onset to admission, missing important data (e.g., disease severity at admission or the primary outcome of disease progression), or severe-to-critical COVID-19 at admission. Additionally, patients who progressed to severe COVID-19 before or on the day of FZJD treatment initiation were also excluded. As the median (interquartile range, IQR) time for FZJD treatment initiation was 3 (1, 10) days, to minimize potential immortal time bias and enhance between-group comparability, we excluded cases in the non-FZJD group that progressed to severe illness within 3 days of hospital admission.

### 2.3 Treatments

Patients were divided into two groups: the FZJD group and the non-FZJD group. The FZJD group included patients who received FZJD granules (for at least 1 day) and standard care. The components and dosages of FZJD are listed in Supplementary Table S2. The non-FZJD group included patients who had received standard care according to China’s *Diagnosis and Treatment Guidelines for COVID-19* [8th (National Health Comission and National Administration of Traditional Chinese Medicine, 2023b) and 9th (National Health Comission and National Administration of Traditional Chinese Medicine, 2023c)] but not FZJD. The standard care included respiratory support, glucocorticoids, convalescent plasma, human COVID-19 immunoglobulin, anticoagulation therapy, and tocilizumab. Based on these treatments, the 8th edition recommended antiviral agents (interferon-α, ribavirin/interferon combinations, lopinavir/ritonavir, chloroquine phosphate, and Arbidol) based on limited early pandemic evidence. The 9th edition discontinued these antiviral recommendations and recommended Paxlovid (nirmatrelvir/ritonavir) and azvudine as first-line antivirals. As remdesivir and molnupiravir are not recommended in the guidelines, none of the patients received either of these treatments.

### 2.4 Primary and secondary outcomes

The primary outcome was the proportion of patients who developed severe-to-critical COVID-19 (hereafter referred to as severe COVID-19) during the hospitalization period. Severe COVID-19 referred to patients who had at least one of the following: (1) respiratory distress (≥30 breaths/min), (2) oxygen saturation ≤93% at rest, (3) arterial partial pressure of oxygen (PaO_2_)/fraction of inspired oxygen (FiO_2_) ≤300 mmHg, or (4) pulmonary lesion progression greater than 50% within 24–48 h. Critical COVID-19 referred to patients who developed respiratory failure and required mechanical ventilation or shock, or who experienced multiple organ dysfunction that necessitated admission to the intensive care unit (ICU). The respiratory rate and blood oxygen level were fully documented on the nursing recording sheet. The secondary outcomes were COVID-19-related mechanical ventilation, inhospital death, and ICU admission.

### 2.5 Statistical analyses

The categorical variables were reported as percentages, whereas the continuous variables were presented as the means (standard deviations, SDs) or medians (IQRs) as appropriate. To minimize selection bias, the independent association between FZJD treatment and disease progression was assessed through propensity score (PS) analyses. Nearest neighbor matching with a caliper width of 0.1 was conducted, with the PS calculated using the variables listed in Table 1. Following PS matching, intergroup differences were evaluated using the standardized mean difference (SMD), with an absolute SMD <0.1 indicating a balance between the groups. Post-matching, univariable logistic regression analysis was performed to investigate associations between FZJD use and clinical outcomes, with the Clopper‒Pearson “exact” method used to calculate the 95% confidence interval (CI) for the proportion of primary and secondary clinical outcomes.

**TABLE 1:** Characteristics of patients with or without FZJD.

Characteristic	Unmatched			Matched		
	FZJD (n = 451)	Non-FZJD (n = 1,193)	SMD	FZJD (n = 320)	Non-FZJD (n = 320)	SMD
Age [years, mean (SD)]	68.3 (13.3)	60.2 (14.2)	0.591	66.0 (13.6)	64.6 (13.3)	0.099
Female (n, %)	262 (58.1)	637 (55.4)	0.094	181 (56.6)	178 (55.6)	0.019
Variants of SARS-CoV-2, (n, %)			0.167			0.050
Omicron variant^a^	346 (76.7)	827 (69.3)		233 (72.8)	240 (75.0)	
Delta variant	105 (23.3)	366 (30.7)		87 (27.2)	80 (25.0)	
Comorbidities, n (%)						
Hypertension	173 (38.4)	413 (34.6)	0.078	122 (38.1)	116 (36.2)	0.039
Heart diseases^b^	22 (4.9)	43 (3.6)	0.063	13 (4.1)	9 (2.8)	0.069
Diabetes	89 (19.7)	180 (15.1)	0.123	58 (18.1)	63 (19.7)	0.040
Chronic lung diseases	24 (5.3)	47 (3.9)	0.066	19 (5.9)	23 (7.2)	0.050
Chronic liver diseases	8 (1.8)	47 (3.9)	0.130	6 (1.9)	11 (3.4)	0.097
Chronic kidney diseases	14 (3.1)	16 (1.3)	0.120	6 (1.9)	3 (0.9)	0.080
Cerebrovascular diseases	39 (8.6)	32 (2.7)	0.260	18 (5.6)	19 (5.9)	0.013
Cancer	12 (2.7)	65 (5.5)	0.142	11 (3.4)	14 (4.4)	0.048
Days from illness onset to admission (median, IQR)	2.0 (1.0–4.0)	1.0 (1.0–3.0)	0.211	2.0 (1.0–4.0)	2.0 (1.0–4.0)	0.005
Vaccination status, n (%)			0.082			0.064
Unvaccinated or 1-dose vaccinated	161 (39.7)	403 (35.7)		132 (41.2)	122 (38.1)	
Fully vaccinated against COVID-19^c^	245 (60.3)	762 (64.3)		188 (58.8)	198 (61.9)	
COVID-19 severity at admission, n (%)			0.533			0.043
Asymptomatic^d^	37 (8.2)	304 (25.5)		34 (10.6)	36 (11.2)	
Mild^e^	294 (65.2)	524 (43.9)		190 (59.4)	194 (60.6)	
Moderate^f^	120 (26.6)	365 (30.6)		96 (30.0)	90 (28.1)	
Concomitant antivirus treatment^g^	147 (32.6)	96 (8.1)	0.641	60 (18.8)	72 (22.5)	0.093

^a^ Including two waves of COVID-19, in which BA.2.38 (n = 224) and BA.5.1.3 (n = 950) of Omicron variant were the dominant variants.

^b^ Heart diseases include coronary heart disease, heart failure, arrhythmias requiring clinical intervention, heart valve disease, and cardiomyopathy.

^c^ Patients who received 2, 3, and 4 doses of any kind of COVID-19 vaccines were defined as having been fully vaccinated.

^d^ Asymptomatic: referred to a positive RT-PCR test without clinical manifestations.

^e^ Mild: defined as mild symptoms without signs of viral pneumonia in the chest computed tomography (CT) images.

^f^ Moderate: referred to patients with fever and respiratory symptoms with radiological manifestations of pneumonia.

^g^ Concomitant antiviral treatment: denoted Paxlovid or azvudine. As of the last patient, remdesivir or molnupiravir was not recommended by the diagnostic and treatment guidelines in mainland China, and no patients were treated with either of these antiviral drugs; therefore, we did not take these two viruses into account.

Missing data for sex, n = 1(0.06%); time from symptom onset to admission, n = 18 (1.10%); fully vaccinated against COVID-19, n = 109 (6.63%). Abbreviations: SARS-CoV-2, severe acute respiratory syndrome coronavirus 2.

The sample size was based on the number of eligible patients at the designated hospitals during the data collection period, as opposed to a formal statistical hypothesis.

### 2.6 Sensitivity analyses

Multiple sensitivity analyses were used to evaluate the robustness of our findings. First, to ensure the robustness of the PSM results for the primary outcome of disease progression, we utilized the estimated PS as a weight and applied three different models: the inverse probability of treatment weighting (IPTW) (Austin and Stuart, 2015), standardized mortality ratio weighting (SMRW) (Desai and Franklin, 2019), and pairwise algorithm (PA) (Li and Greene, 2013). These models generated a weighted cohort, thus allowing for the assessment of independent associations between FZJD use and disease progression through a logistic regression analysis. Additionally, we also performed multivariable logistic regression using the whole cohort to calculate the adjusted odds ratio (OR). Furthermore, we performed an additional multivariable logistic regression analysis to address missing data by employing multiple imputations with five replications and a chained equation approach, using the R mice package. Second, potential unmeasured confounders between FZJD use and disease progression were evaluated using the *E*-value (VanderWeele and Ding, 2017), which quantifies the degree of unmeasured confounding factors that may offset the effect of FZJD on disease progression. Third, logistic regression analysis after PSM was used to explore the association of disease progression with FZJD in different subgroups. The groups were categorized according to sex, older age (≥60 years), COVID-19 severity at admission, vaccination status, and the presence of comorbidities. We assessed interactions between FZJD treatment and subgroups. Fourth, to minimize potential immortal time bias, we further excluded 55 patients who progressed to severe illness within 5 days of hospital admission among non-FZJD users. The majority (63.95%) of patients in this group experienced disease progression during this period. Notably, this exclusion may underestimate the effectiveness of FZJD.

All of the analyses were performed using R (v.3.3.2; R Foundation for Statistical Computing, Vienna, Austria; http://www.R-project.org, accessed on 1 January 2021), SPSS 18.0, and the Free Statistics Analysis Platform (v.1.8), with a two-sided *P* value <0.05 considered to be statistically significant.

## 3 Results

### 3.1 Demographic characteristics

**FIGURE 1:**
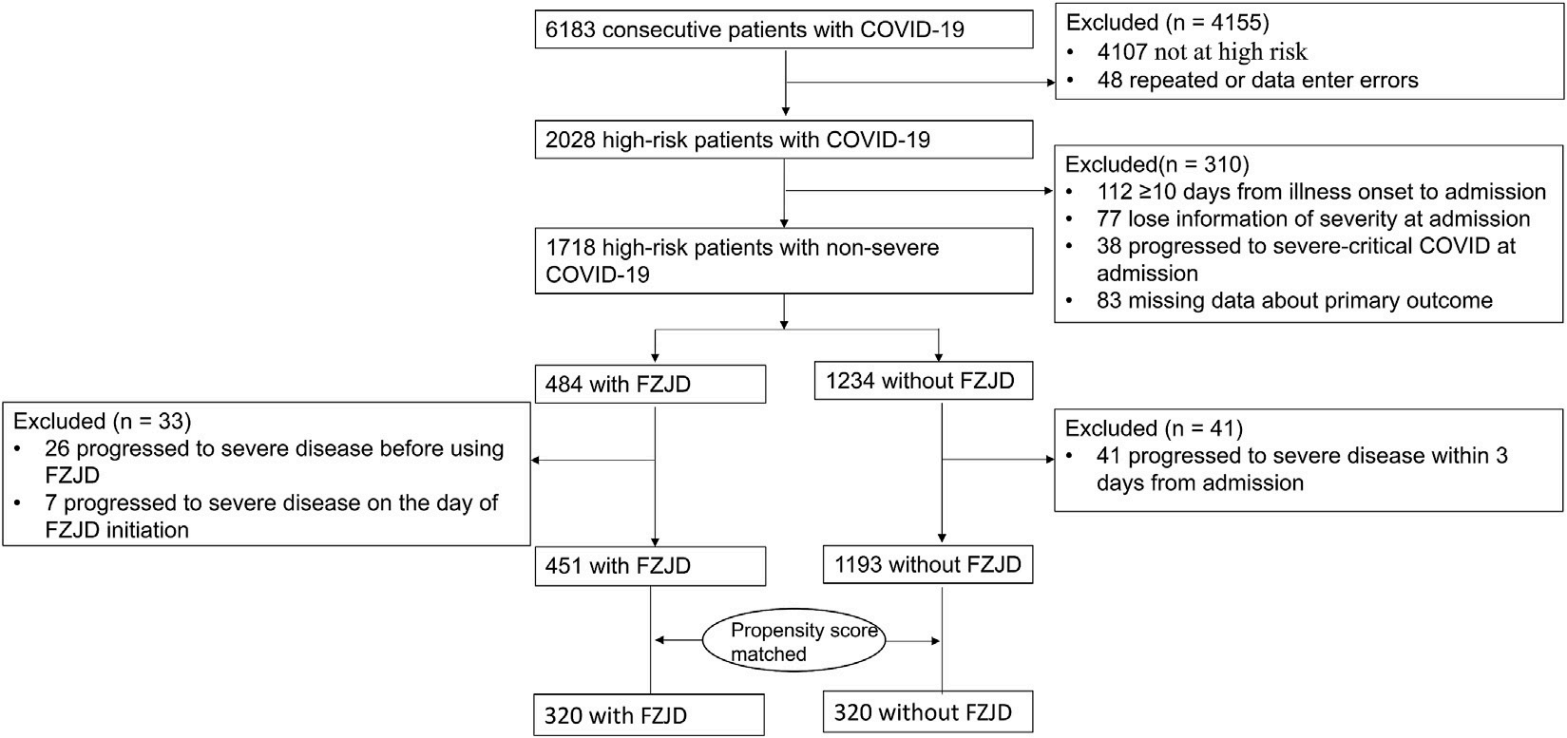
Flowchart of the study.

Out of the 6,183 COVID-19 patients who were admitted to designated hospitals, 4,155 were excluded because they were not at high risk (n = 4107) or had repeated data (n = 48). Among the remaining 2,028 high-risk patients, 343 were further excluded because of (1) delayed admission beyond 10 days from illness onset (n = 112), (2) progression to severe COVID-19 at admission (n = 38), (3) missing data of severity at admission (n = 77) or primary outcomes (n = 83), and (4) progression to severe COVID-19 before or on the day of FZJD initiation (n = 33). To avoid immortal time bias, 41 patients who progressed to severe disease within 3 days of admission in the non-FZJD group were also excluded (accounting for 48.2% of those who progressed to severe disease in the non-FZJD group). Ultimately, the analysis included 1,644 patients, among whom 451 (27.4%) received FZJD and 1,193 (72.6%) did not receive FZJD (Figure 1).

Among the included patients, female patients accounted for 54.7%. The mean (SD) age was 62.3 (14.5) years. As all COVID-19 patients in one area were treated in one designated hospital during the study period, no patients were transferred. The median time between illness onset and hospital admission was 1 day, and the median duration of FZJD use was 7 days. Table 1 describes the baseline characteristics of the patients in the unmatched and PS-matched cohorts. In the unmatched cohort, FZJD exposure differed according to age, infected variants of SARS-CoV-2, comorbidities (diabetes, chronic liver diseases, chronic kidney diseases, cerebrovascular diseases, and cancer), the time between illness onset and admission, COVID-19 severity at admission, and concomitant antivirus treatments (SMD >0.1). After matching, 320 patients received FZJD and 320 patients did not receive FZJD. The characteristics were well balanced between the two groups in the matched cohort (SMD <0.1) (Supplementary Figure S1).

### 3.2 Primary outcomes

**FIGURE 2:**
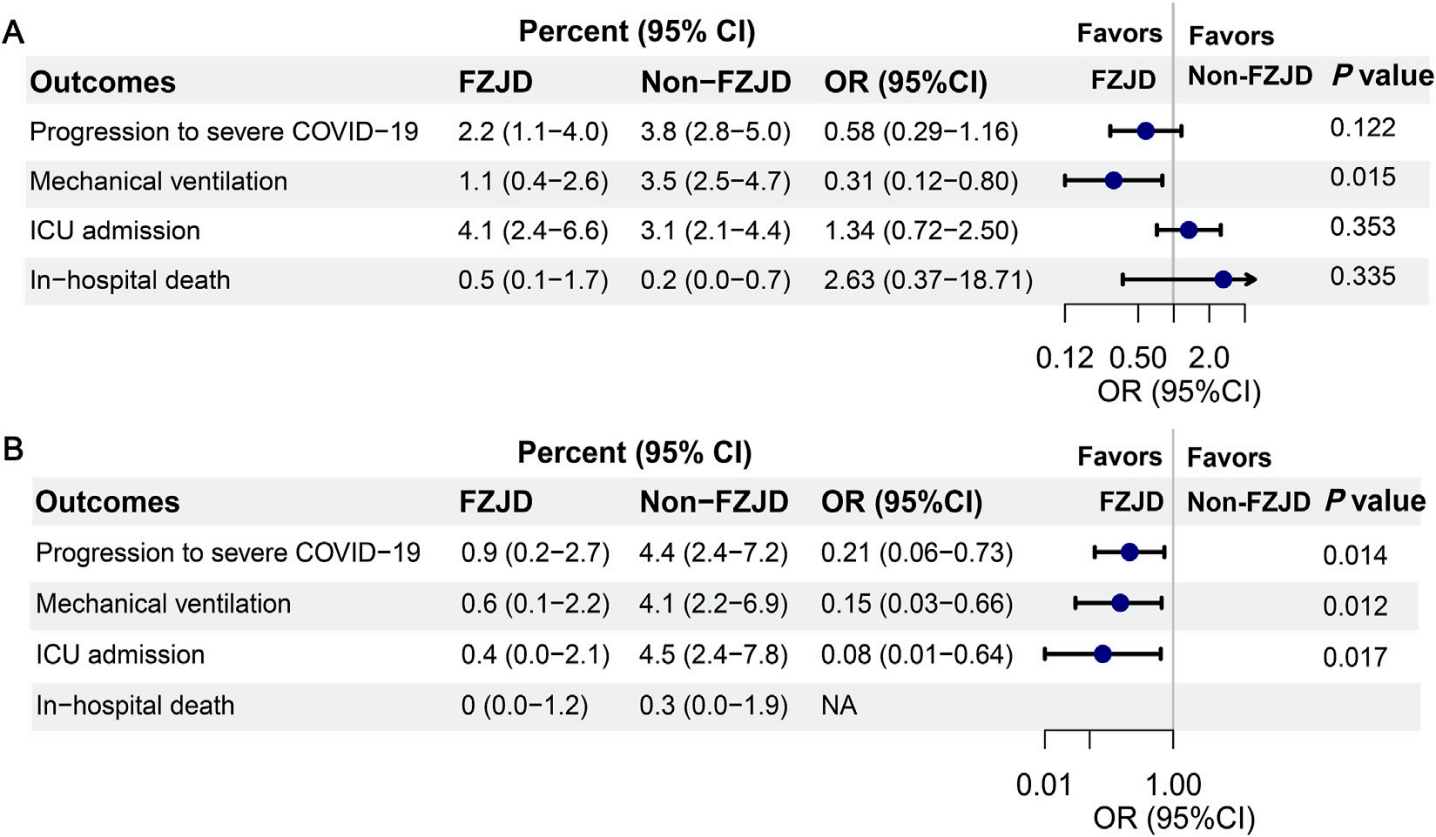
Clinical outcomes of patients with or without FZJD. **(A)** Clinical outcomes of unmatched patients. **(B)** Clinical outcomes of matched patients. It should be noted that patients may be in more than one category (e.g., progression to severe COVID-19 and mechanical ventilation). Mechanical ventilation included high-flow nasal cannula oxygen therapy, noninvasive mechanical ventilation, and invasive mechanical ventilation. The 95% CI percent was calculated using the Clopper–Pearson exact method. Abbreviations: ICU, intensive care unit; CI, confidence interval.

Among the 1,644 patients who were analyzed, 3.3% (55 individuals) progressed to severe disease, with fewer cases observed in FZJD users than in nonusers (as shown in Figure 2A, 2.2% vs. 3.8%, *P* = 0.122). After PSM, the proportion of disease progression was significantly lower in FZJD users than in the nonusers (0.9% vs. 4.4%, *P* = 0.014, Figure 2B). The absolute risk reduction in the matched cohort was 3.5%, which indicated that the number needed to treat (NNT) was 28.6.

We excluded cases that progressed to severe COVID-19 within 3 days in non-FZJD users and then calculated the ORs. As shown in Table 2 and Figure 2, the PSM analysis indicated a reduced risk of disease progression in the FZJD group (OR, 0.21; 95% CI, 0.06–0.73). The result was further supported by four additional PS analyses (PS adjustment, IPTW, SMRW, and PA models) and multivariable logistic regression analyses (ORs ranging from 0.17 to 0.40 and all *P*-values <0.05). Moreover, the *E*-value for this cohort ranged from 4.436 to 11.241 (Supplementary Table S3). To further avoid immortal time bias, additional sensitivity analyses were conducted after excluding cases that progressed to severe COVID-19 within 5 days in non-FZJD users (n = 55, accounting for 63.95% of those progressed to severe disease in the non-FZJD group) (Supplementary Table S4). The results were similar to those of the main analysis (ORs ranging from 0.22 to 0.44 and all *P*-values <0.05; *E*-value for this cohort ranged from 3.973 to 8.560; Supplementary Table S5).

**TABLE 2:** Associations between treatment with FZJD and disease progression in the crude, multivariable, and propensity-score analyses.

Analysis-OR (95% CI)	FZJD (N)	Non-FZJD (N)	Progression to severe disease-OR (95% CI)	*P*-value
Crude analysis	451	1,193	0.35 (0.14–0.88)	0.026
Multivariable analysis^a^	451	1,193	0.23 (0.09–0.62)	0.004
Multivariable analysis after MI^b^	451	1,193	0.40 (0.19–0.85)	0.017
Propensity-score analysis				
Adjusted for propensity score^c^	451	1,193	0.21 (0.07–0.58)	0.003
With matching^d^	320	320	0.21 (0.06–0.73)	0.014
With IPTW^e^	451	1,193	0.19 (0.06–0.62)	0.006
With SMRW^e^	451	1,193	0.30 (0.12–0.75)	0.011
With PA^e^	451	1,193	0.17 (0.05–0.63)	0.008

^a^ Sensitivity analyses, the OR was calculated using multivariable logistic regression analysis adjusted for age, gender, presence of comorbidities, disease severity at admission, and vaccination status. We adjusted only for these five covariates in the logistic regression model due to the relatively small number of events (n = 55).

^b^ Sensitivity analyses, the OR was calculated using multivariable logistic regression analysis after multiple imputations adjusted for age, gender, presence of comorbidities, disease severity at admission, and vaccination status using data from the entire cohort.

^c^ Sensitivity analyses, the OR was calculated using multivariable logistic regression adjusted for the same strata and covariates with matching and additionally adjusted for the propensity score.

^d^ Primary analysis, the OR was calculated after PSM. Matched by covariates listed in Table 1.

^e^ Sensitivity analyses, the OR was calculated using the multivariable logistic regression with the same strata and covariates with IPTW, SMRW, and PA models according to the propensity score. Matched by the same covariates listed for PSM.

Abbreviations: MI, multiple imputation; IPTW, inverse probability of treatment weighting; SMRW, standardized mortality ratio weighting; PA, pairwise algorithmic.

### 3.3 Secondary outcomes

The proportion of mechanical ventilation was significantly lower in FZJD users than in the nonusers in the matched cohort (0.6% vs. 4.1%, *P* = 0.012, Figure 2B). Similar results were found in the proportion of ICU admission (0.4% vs. 4.5%, *P* = 0.017, Figure 2B). No significant difference was observed in the inhospital mortality rate in the unmatched cohorts (Figure 2A). After matching, there were no deaths in the FZJD group, whereas one patient in the non-FZJD group experienced mortality (Figure 2B).

### 3.4 Subgroup analyses

**FIGURE 3:**
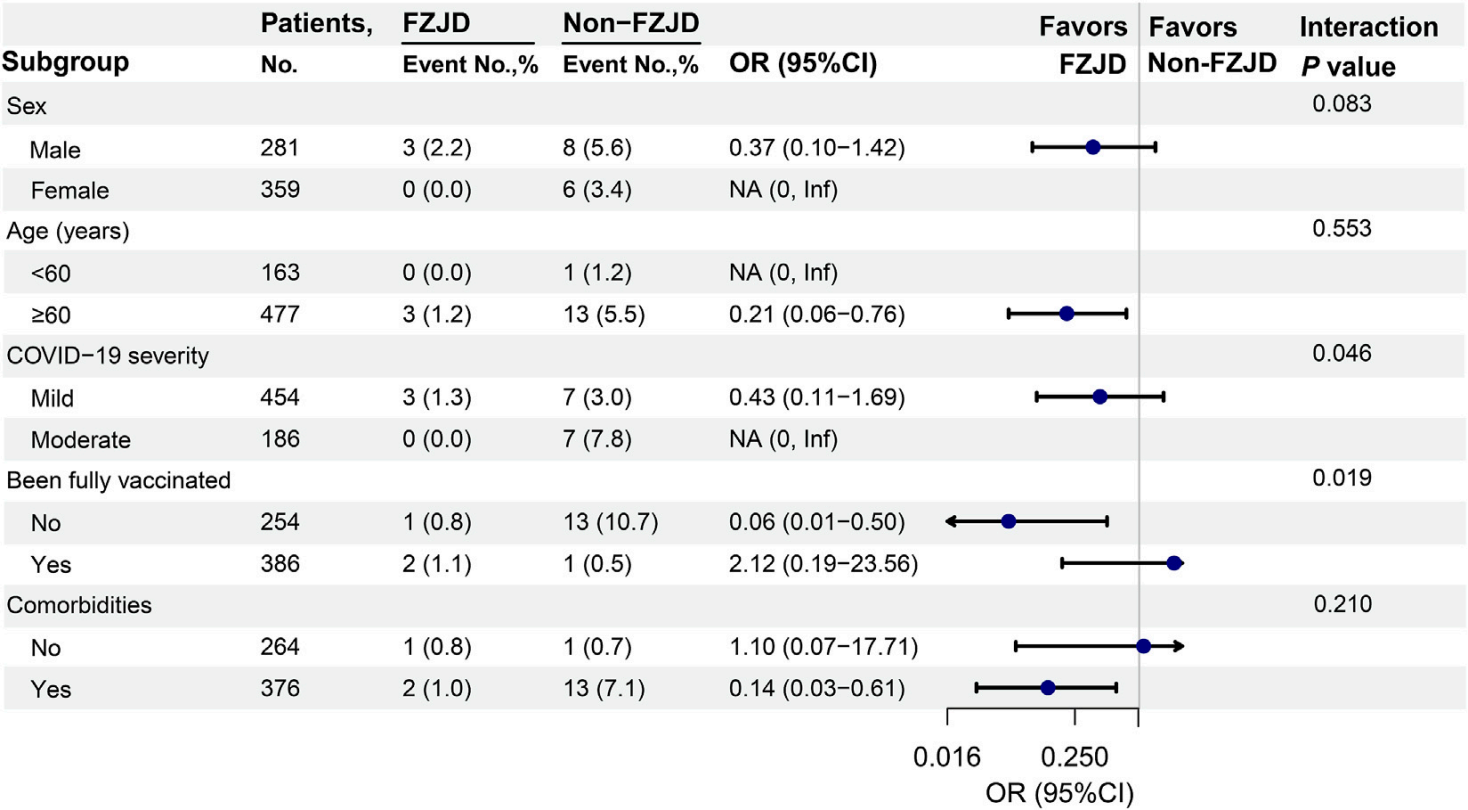
Associations between treatment with FZJD and disease progression in different subgroups. NA: OR and P-value could not be calculated as no patient progressed to severe status. COVID-19 severity at admission: “mild” included asymptomatic and mild COVID-19. Been fully vaccinated against COVID-19: “no” included unvaccinated and 1-dose vaccinated, and “yes” included patients who received 2, 3, or 4 doses of any kind of COVID-19 vaccines. Abbreviation: Inf, infinite.

Logistic regression analyses were conducted to assess disease progression risk in different subgroups, as described in Figure 3. Interaction tests demonstrated significant heterogeneity in COVID-19 severity at admission and vaccination status. Specifically, patients with moderate COVID-19 or those who were not fully vaccinated showed a significant treatment effect. The trend of treatment benefit was observed only in elderly patients (OR, 0.21; 95% CI, 0.06–0.76) and patients with comorbidities (OR, 0.14; 95% CI, 0.03–0.61).

## 4 Discussion

In this real-world study of high-risk patients with non-severe COVID-19, we first demonstrated that FZJD was associated with a lower risk of disease progression (OR, 0.21; 95% CI, 0.06–0.73 with PSM). These results remained consistent across various sensitivity analyses. Notably, the subgroup analysis demonstrated significant treatment heterogeneity. Pronounced benefits were observed in patients exhibiting moderate COVID-19, older adults, patients with comorbidities, or patients who were not fully vaccinated. Our findings may provide new evidence and insights into the treatment of COVID-19.

The protective effectiveness of FZJD against disease progression may be attributed to its dual mechanisms: reducing excessive inflammatory responses (Lu et al., 2022; Li et al., 2025; Chen et al., 2023; Lu et al., 2024; Li et al., 2021) and restoring the integrity of the pulmonary epithelial barrier (Chen et al., 2023). Specifically, treatment with FZJD has been shown to significantly downregulate key inflammatory mediators such as IL-1β, IL-6, TNF-α, IL-13, IL-17, CCL2, CXCL9, and CXCL10, which played a crucial role in the development of cytokine storms (Li et al., 2025). This suggested that FZJD effectively suppressed hyperinflammatory cascades, thereby alleviating severe pulmonary tissue damage. Mechanistically, FZJD inhibited the activation of the NLRP3 inflammasome (Li et al., 2025) and modulated the amino acid metabolism via the gut–microbiota–lung axis (Lu et al., 2024). Additionally, FZJD-containing serum has been demonstrated to significantly mitigate M1 macrophage polarization and suppress the production of reactive oxygen species (ROS) by blocking the PI3K/Akt pathway while simultaneously enhancing the integrity of the alveolar epithelial barrier in a co-culture system of LPS-induced alveolar macrophages and type II alveolar epithelial cells (Chen et al., 2023). These coordinated mechanisms stabilized pulmonary histoarchitecture, thus impeding disease progression.

Concomitant treatments may bias the results. In this study, both groups received standard care according to China’s COVID-19 guidelines (8th edition, August 2020; 9th edition, March 2022). The 8th edition recommended antiviral therapies with limited early pandemic evidence such as interferon-α, combinations of ribavirin and interferon, lopinavir/ritonavir, chloroquine phosphate, and Arbidol. In contrast, the 9th edition discontinued these antiviral recommendations and instead endorsed Paxlovid (nirmatrelvir/ritonavir) and azvudine as first-line antivirals with proven efficacy (Hammond et al., 2022; Yang et al., 2023) in preventing disease progression among high-risk populations. The two antiviral agents were included as matching covariates during PSM to mitigate confounding factors. The post-PSM analysis indicated a balanced allocation between groups, with a standardized mean difference of less than 0.1, which confirmed that the allocation of antiviral treatments was comparable. However, remdesivir and molnupiravir, though potentially efficacious, were excluded from matching due to their absence from national guidelines and clinical use.

Other TCM formulas, such as the Hanshiyi Formula (Tian et al., 2020), NRICM101, and NRICM102 (Tseng et al., 2022), presented benefits in reducing the rate of conversion to severe cases in the general population. Different from these formulas, FZJD contains Yang-warming herbs, including Danfupian (*Aconiti Lateralis Radix Praeparata*), Ganjiang (*Zingiberis Rhizoma*), Zhigancao (*Glycyrrhizae Radix Et Rhizoma*), and Wuzhimaotao (*Fici Radix*), targeting high-risk patients with “Yang Qi Deficiency and Lingering Dampness-Toxin” syndrome. Our subgroup analyses revealed pronounced therapeutic benefits in key vulnerable subgroups: patients with moderate COVID-19, older adults, patients with comorbidities, and incompletely vaccinated individuals. This finding may be especially significant for individuals who are unable to receive COVID-19 vaccination due to contraindications (e.g., those who are severely immunocompromised, pregnant, or have vaccine allergies). As older age, comorbidities, and moderate COVID-19 increase the risk of mortality (Ko et al., 2021; Zheng et al., 2020), the potential of FZJD in promoting recovery for these patients is significant.

### 4.1 Strengths and limitations

There are several limitations in the present study. First, incomplete data on tobacco use (40.4%) and body mass index [BMI] (49.2%) precluded their inclusion as confounders. Second, although PSM rigorously adjusted for baseline characteristics, including demographics, comorbidities, the time between illness onset and admission, COVID-19 severity at admission, vaccination status, and concomitant antiviral therapies, residual confounding by unmeasured factors (e.g., BMI) cannot be entirely excluded. To quantify the potential impact of such omissions, we conducted E-value sensitivity analyses. The E-values for our primary outcomes ranged from 4.436 to 11.241, indicating that an unmeasured confounder would need to exhibit implausibly strong associations with both treatment exposure and clinical outcomes [risk ratios (RR) >4.436–11.241] to fully negate the observed effects, underscoring the robustness of our conclusions against potential residual bias. Third, the detection of viral RNA loads and viral antigens was different across the six hospitals, and the comparability of viral RNA loads was compromised; thus, we were unable to assess the effectiveness of FZJD based on the change in the viral load.

Despite the limitations of the current study, there were some notable strengths. First, this represents the first large-scale study providing rigorous effectiveness evaluation of this formula since its market approval. Second, during the study phase, due to China’s “dynamic zero-COVID” policy, all of the patients in our study were primarily infected, thereby minimizing the impact of prior infections on treatment effectiveness evaluation. Third, the results withstood a range of sensitivity analyses, which adjusted for possible confounders. The results were still consistent when excluding cases that progressed to severe COVID-19 within 5 days in non-FZJD users. Through several analyses, we confirmed the independent association between FZJD treatment and a reduced risk of disease progression. Furthermore, our multicenter study design increased the generalizability of our findings.

## 5 Conclusion

FZJD combined with standard care was associated with a significantly lower risk of disease progression. Further randomized controlled trials are needed to validate our results.

## Data Availability

The raw data supporting the conclusions of this article will be made available by the authors, without undue reservation.

## Abbreviations

COVID-19, coronavirus disease; FZJD, Fuzheng Jiedu; PSM, propensity score matching; OR, odds ratio; CI, confidence interval; SARS-CoV-2, severe acute respiratory syndrome coronavirus 2; CHM, Chinese herbal medicine; SD, standard difference; IQR, interquartile range; PS, propensity score; SMD, standardized mean difference; IPTW, inverse probability of treatment weighting; SMRW, standardized mortality ratio weighting; PA, pairwise algorithmic; UHPLC-Q-TOF/MS, ultra-high-performance liquid chromatography coupled with quadrupole time-of-flight mass spectrometry; BMI, body mass index.

## References

[B1] Austin P. C. Stuart E. A. (2015). Moving towards best practice when using inverse probability of treatment weighting (IPTW) using the propensity score to estimate causal treatment effects in observational studies. Stat. Med. 34, 3661–3679. 10.1002/sim.6607 26238958 PMC4626409

[B2] Bernal A. J. Silva M. M. G. da Musungaie D. B. Kovalchuk E. Gonzalez A. Reyes V. D. (2021). Molnupiravir for oral treatment of covid-19 in nonhospitalized patients. N. Engl. J. Med. 386, 509–520. 10.1056/NEJMoa2116044 34914868 PMC8693688

[B3] Chen J. Lu Y. Li J. Zhang Z. Liu Y. Yang R. (2023). Study on the inflammatory regulation of serum containing Fuzheng Jiedu Recipe on the co-culture of mouse alveolar macrophages and type II alveolar epithelial cells. Lishizhen Med. Materia Medica Res. 34, 554–558. (in Chinese). 10.3969/j.issn.1008-0805.2023.03.11

[B4] Chen Y. Wei D. Zhao J. Xu X. Chen J. (2021). Reduction of hyperoxic acute lung injury in mice by Formononetin. PLOS ONE 16, e0245050. 10.1371/journal.pone.0245050 33411783 PMC7790402

[B5] Desai R. J. Franklin J. M. (2019). Alternative approaches for confounding adjustment in observational studies using weighting based on the propensity score: a primer for practitioners. BMJ 367, l5657. 10.1136/bmj.l5657 31645336

[B6] Dougan M. Nirula A. Azizad M. Mocherla B. Gottlieb R. L. Chen P. (2021). Bamlanivimab plus etesevimab in mild or moderate covid-19. N. Engl. J. Med. 385, 1382–1392. 10.1056/NEJMoa2102685 34260849 PMC8314785

[B7] Gottlieb R. L. Vaca C. E. Paredes R. Mera J. Webb B. J. Perez G. (2022). Early Remdesivir to prevent progression to severe covid-19 in outpatients. N. Engl. J. Med. 386, 305–315. 10.1056/NEJMoa2116846 34937145 PMC8757570

[B8] Hammond J. Leister-Tebbe H. Gardner A. Abreu P. Bao W. Wisemandle W. (2022). Oral nirmatrelvir for high-risk, nonhospitalized adults with covid-19. N. Engl. J. Med. 386, 1397–1408. 10.1056/NEJMoa2118542 35172054 PMC8908851

[B9] Hosawi S. (2023). Current update on role of Hesperidin in inflammatory lung diseases: chemistry, pharmacology, and drug delivery approaches. Life. 13, 937. 10.3390/life13040937 37109466 PMC10145343

[B10] Huang K. Zhang P. Zhang Z. Youn J. Wang C. Zhang H. (2021). Traditional Chinese Medicine (TCM) in the treatment of COVID-19 and other viral infections: efficacies and mechanisms. Pharmacol. Ther. 225, 107843. 10.1016/j.pharmthera.2021.107843 33811957 PMC8011334

[B11] Huang Y. Wang W. Liu Y. Wang Z. Cao B. (2024). COVID-19 vaccine updates for people under different conditions. Sci. China Life Sci. 67, 2323–2343. 10.1007/s11427-024-2643-1 39083202

[B12] Ko J. Y. Danielson M. L. Town M. Derado G. Greenlund K. J. Kirley P. D. (2021). Risk factors for coronavirus disease 2019 (COVID-19)–Associated hospitalization: COVID-19–Associated hospitalization surveillance network and behavioral risk factor surveillance system. Clin. Infect. Dis. 72, e695–e703. 10.1093/cid/ciaa1419 32945846 PMC7543371

[B13] Li G. Li Y. Liu Y. Xi X. Zhang Z. (2021). Potential mechanism prediction of Fuzheng Jiedu Granules against COVID-19 via network pharmacology analysis and molecular docking, Chin. Front. Health Quar. 44, 311–318. (in Chinese). 10.16408/j.1004-9770.2021.05.003

[B14] Li L. Greene T. (2013). A weighting analogue to pair matching in propensity score analysis. Int. J. Biostat. 9, 215–234. 10.1515/ijb-2012-0030 23902694

[B15] Li Y. Zou H. Ma L. Hu D. Long H. Lin J. (2025). Fuzheng Jiedu decoction alleviates H1N1 virus-induced acute lung injury in mice by suppressing the NLRP3 inflammasome activation. J. Ethnopharmacol. 341, 119314. 10.1016/j.jep.2024.119314 39746408

[B16] Liu Y. Zhang T. Zhu W. Xi X. Guo J. Zou X. (2022). Protective Effect and Mechanism of Fuzheng Jiedu Granules on Lipopolysaccharide-induced Acute Lung Injury in Mice. Tradit Chin Drug Res Clin Pharmacol. 33:588. (in Chinese). 10.19378/j.issn.1003-9783.2022.05.004

[B17] Lu Y. Wu Y. Huang M. Chen J. Zhang Z. Li J. (2024). Fuzhengjiedu formula exerts protective effect against LPS-induced acute lung injury via gut-lung axis. Phytomedicine 123, 155190. 10.1016/j.phymed.2023.155190 37972468

[B18] National Health Comission and National Administration of Traditional Chinese Medicine (2023a). Diagnosis Treat. COVID-19 (trial version 10). Available online at: https://www.gov.cn/zhengce/zhengceku/2023-01/06/content_5735343.htm (Accessed May 13, 2023).

[B19] National Health Comission and National Administration of Traditional Chinese Medicine (2023b). Diagnosis and treatment of COVID-19 (trial version 8). Available online at: https://www.gov.cn/zhengce/zhengceku/2020-08/19/content_5535757.htm (Accessed May 13, 2023).

[B20] National Health Comission and National Administration of Traditional Chinese Medicine (2023c). Diagnosis and treatment of COVID-19 (trial version 9). Available online at: https://www.gov.cn/zhengce/zhengceku/2022-03/15/content_5679257.htm (Accessed May 13, 2023).

[B21] National Medical Products Administration (2025). Search results from the national medical products administration (NMPA) Available online at: https://www.nmpa.gov.cn/datasearch/search-info.html?nmpa=aWQ9MTQ5NjM1YzBkYmMzZjA1NmI3MzA5MTJlMDEzZWEyYWMmaXRlbUlkPWZmODA4MDgxODNjYWQ3NTAwMTg0MDg4MWY4NDgxNzlm (Accessed March 3, 2025).

[B22] Shi N. Liu B. Liang N. Ma Y. Ge Y. Yi H. (2020). Association between early treatment with Qingfei Paidu decoction and favorable clinical outcomes in patients with COVID-19: a retrospective multicenter cohort study. Pharmacol. Res. 161, 105290. 10.1016/j.phrs.2020.105290 33181320 PMC7833425

[B23] Shu Z. Chang K. Zhou Y. Peng C. Li X. Cai W. (2021). Add-on Chinese medicine for coronavirus disease 2019 (accord): a retrospective cohort study of hospital registries. Am. J. Chin. Med. 49, 543–575. 10.1142/S0192415X21500257 33683189

[B24] Tian J. Yan S. Wang H. Zhang Y. Zheng Y. Wu H. (2020). Hanshiyi Formula, a medicine for Sars-CoV2 infection in China, reduced the proportion of mild and moderate COVID-19 patients turning to severe status: a cohort study. Pharmacol. Res. 161, 105127. 10.1016/j.phrs.2020.105127 32791263 PMC7416080

[B25] Tseng Y.-H. Lin S. J.-S. Hou S.-M. Wang C.-H. Cheng S.-P. Tseng K.-Y. (2022). Curbing COVID-19 progression and mortality with traditional Chinese medicine among hospitalized patients with COVID-19: a propensity score-matched analysis. Pharmacol. Res. 184, 106412. 10.1016/j.phrs.2022.106412 36007774 PMC9395232

[B26] VanderWeele T. J. Ding P. (2017). Sensitivity analysis in observational research: introducing the E-value. Ann. Intern Med. 167, 268–274. 10.7326/M16-2607 28693043

[B27] Wang Y. Liu Y. Lv Q. Zheng D. Zhou L. Ouyang W. (2021). Effect and safety of Chinese herbal medicine granules in patients with severe coronavirus disease 2019 in Wuhan, China: a retrospective, single-center study with propensity score matching. Phytomedicine 85, 153404. 10.1016/j.phymed.2020.153404 33637412 PMC7642753

[B28] Yang H. Wang Z. Jiang C. Zhang Y. Zhang Y. Xu M. (2023). Oral azvudine for mild‐to‐moderate COVID‐19 in high risk, nonhospitalized adults: results of a real‐world study. J. Med. Virol. 95, e28947. 10.1002/jmv.28947 37470209

[B29] Yang L. Nie H. Du Y. Liu X. Cai B. Li J. (2025). Isoliquiritigenin exhibits anti‐inflammatory responses in acute lung injury by covalently binding to the myeloid differentiation protein‐2 domain. Phytother. Res. 39, 922–937. 10.1002/ptr.8411 39697044

[B30] Yang Y. Islam M. S. Wang J. Li Y. Chen X. (2020). Traditional Chinese medicine in the treatment of patients infected with 2019-new coronavirus (SARS-CoV-2): a review and perspective. Int. J. Biol. Sci. 16, 1708–1717. 10.7150/ijbs.45538 32226288 PMC7098036

[B31] Zheng Z. Peng F. Xu B. Zhao J. Liu H. Peng J. (2020). Risk factors of critical and mortal COVID-19 cases: a systematic literature review and meta-analysis. J. Infect. 81, e16–e25. 10.1016/j.jinf.2020.04.021 PMC7177098 32335169

[B32] Zhou H. Yang T. Lu Z. He X. Quan J. Liu S. (2023). Liquiritin exhibits anti-acute lung injury activities through suppressing the JNK/Nur77/c-Jun pathway. Chin. Med. 18, 35. 10.1186/s13020-023-00739-3 37013552 PMC10068703

