# Supplementary material for: Fuzheng Jiedu granules against disease progression among high-risk adults with non-severe COVID-19: a multicenter retrospective cohort study
Source: Front Pharmacol. 2025 May 12;16:1523004. doi: 10.3389/fphar.2025.1523004 (PMC12104657; doi:10.3389/fphar.2025.1523004)
Supplement: Supplementary file 1 [file DataSheet1.docx]

**Supplementary Appendix**

Supplementary information for

**Fuzheng Jiedu granules against disease progression among high-risk adults with non-severe COVID-19: a multicentre retrospective cohort study**


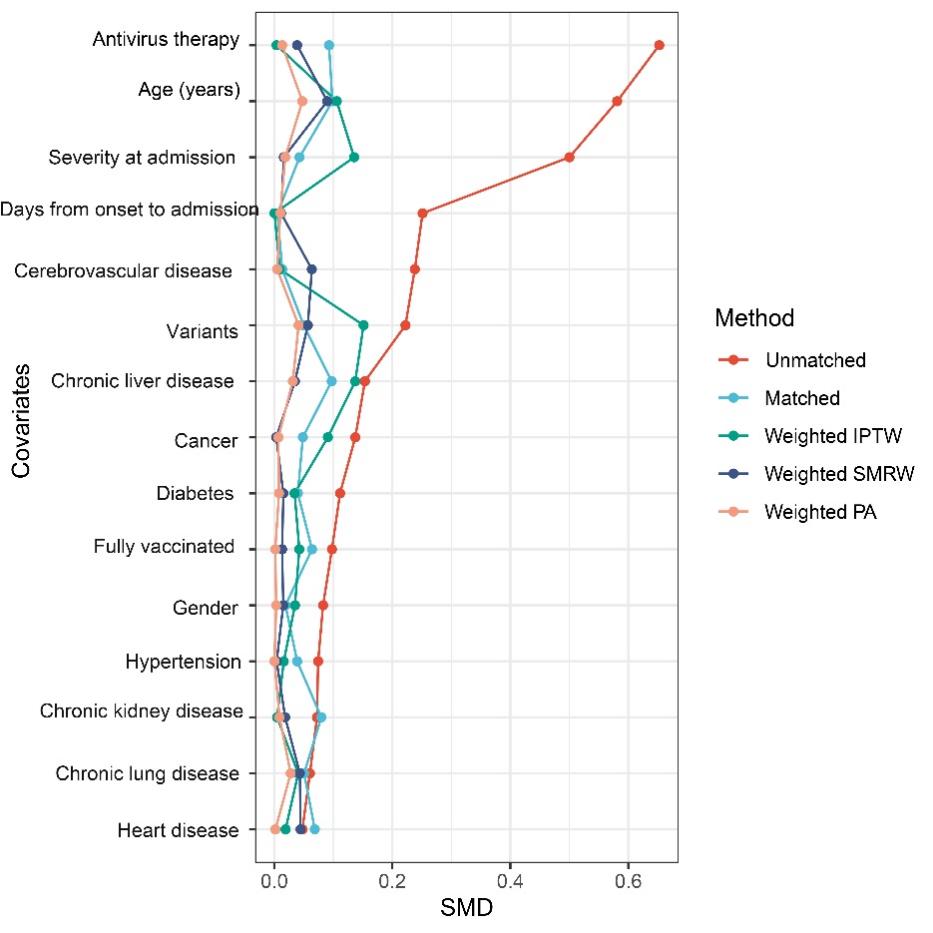


**Fig.S.1.** SMD in the unmatched and other four propensity score analyses.

Abbreviations: IPTW, inverse probability of treatment weighting; SMRW, standardized mortality ratio weighting; PA, pairwise algorithmic.

Within the unmatched cohort, the standardized mean differences (SMD) for several covariates exceeded 0.1. However, after applying propensity score matching, weighted IPTW, weighted SMRW, and weighted PA, the covariates between the FZJD and Non-FZJD groups were well-balanced.

Table S.1 Detailed information of the six designated COVID-19 hospitals

| Hospital names | Hospital level | Sample included | Time period (Different waves of COVID-19) |
| --- | --- | --- | --- |
| Guangzhou Eighth People’s Hospital of Guangzhou Medical University | Teritary A | 57 | 2021-05-21 to 2021-07-07 |
| The First Affiliated Hospital of Xiamen University | Teritary A | 77 | 2021-09-13 to 2021-10-26 |
| Tangshan Campus of the Second Hospital of Nanjing | Teritary A | 318 | 2021-07-21 to 2021-09-12 |
| Dalian Public Health Centre | Teritary A | 18 | 2021-11-05 to 2021-12-14 |
| The Second People's Hospital of Lanzhou | Teritary A | 224 | 2022-07-11 to 2022-08-23 |
| Sanya central hospital | Teritary A | 950 | 2022-08-02 to 2022-09-30 |

Table S.2 The components and dosage of Fuzheng Jiedu Granules (FZJD)

| Chinese names | English names | Latin names | Dosage(g) |
| --- | --- | --- | --- |
| 淡附片 | *Danfupian* | *Aconiti Lateralis Radix Praeparata* | 10 |
| 干姜 | *Ganjiang* | *Zingiberis Rhizoma* | 15 |
| 炙甘草 | *Zhigancao* | *Glycyrrhizae Radix Et Rhizoma* | 20 |
| 金银花 | *Jinyinhua* | *Lonicerae Japonicae Flos* | 10 |
| 皂角刺 | *Zaojiaoci* | *Gleditsiae Spina* | 10 |
| 五指毛桃 | *Wuzhimaotao* | *Fici Radix* | 20 |
| 广藿香 | *Guanghuoxiang* | *Pogostemonis Herba* | 10 |
| 陈皮 | *Chenpi* | *Citri Reticulatae Pericarpium* | 5 |

Administration: all the granules were administered orally three times a day in 100-200 mL warm boiled water. All herbs were industrially extracted into granules by Guangzhou Chinese Medicine University Technology Industry Park Co., Ltd. Therefore, the composition of FZJD granules administered to patients across different units was consistent.

Table S.3 E-values for effectiveness of FZJD after excluding cases progressed to severe COVID-19 within 3 days in Non-FZJD users

| Analyses | E value **^a^** | Lower ^b^ |
| --- | --- | --- |
| Crude analysis | 5.161 | 1.530 |
| Multivariable analysis | 8.163 | 2.607 |
| Multivariable analysis after MI | 4.436 | 1.632 |
| Propensity score analyses |  |  |
| Adjusted for propensity score | 8.994 | 2.842 |
| With matching | 8.994 | 2.082 |
| With IPTW | 10.000 | 2.607 |
| With SMRW | 6.122 | 2.000 |
| With PA | 11.241 | 2.553 |

**^a^** Results are E-values for the adjusted ORs in **Table 2**

^b^ Results are E-values for the upper bound of the CI of adjusted OR

Abbreviations: MI, multiple imputation; IPTW, inverse probability of treatment weighting; SMRW, standardized mortality ratio weighting; PA, pairwise algorithmic.

E-value ranged from 4.436 to 11.241, indicating that strong concurrent confounders are required to change the observed OR.

Table S.4 Associations between treatment with FZJD and disease progression after excluding cases progressed to severe COVID-19 within 5 days in Non-FZJD users.

| Analyses-OR (95% CI) | FZJD (N) | Non-FZJD (N) | Progression to severe disease  OR (95% CI) | *P*-value |
| --- | --- | --- | --- | --- |
| Crude analysis | 451 | 1179 | 0.51 (0.19-1.32) | 0.165 |
| Multivariable analysis ^a^ | 451 | 1179 | 0.26 (0.09-0.71) | 0.021 |
| Multivariable analysis after MI ^b^ | 451 | 1179 | 0.44 (0.20-0.96) | 0.039 |
| Propensity-score analyses |  |  |  |  |
| Adjusted for propensity score ^c^ | 451 | 1179 | 0.25 (0.09-0.73) | 0.011 |
| With matching ^d^ | 323 | 323 | 0.24 (0.07-0.87) | 0.030 |
| With IPTW ^e^ | 451 | 1179 | 0.27 (0.08-0.88) | 0.031 |
| With SMRW ^e^ | 451 | 1179 | 0.37 (0.15-0.96) | 0.040 |
| With PA ^e^ | 451 | 1179 | 0.22 (0.06-0.83) | 0.025 |

^a^ Sensitivity analyses, the OR was calculated using multivariable logistic regression analysis adjusted for age, gender, presence of comorbidities, and vaccination status. we adjusted only for these five covariates in the logistic regression model due to the relatively small number of events (n=41) in the cohort.

^b^ Sensitivity analyses, the OR was calculated using multivariable logistic regression analysis after multiple imputation adjusted for age, sex, presence of comorbidities, and vaccination status using data from the entire cohort.

^c^ Sensitivity analyses, the OR was calculated using multivariable logistic regression adjusted for the same strata and covariates with matching and additionally adjusted for propensity score.

^d^ Primary analysis, the OR was calculated after PSM. nearest neighbor matching with a caliper width of 0.2 was conducted with PS calculated using the variables listed in Table 1.

^e^ Sensitivity analyses, the OR was calculated using the multivariable logistic regression with the same strata and covariates with IPTW, SMRW, and PA model according to the propensity score. Matched by the same covariates listed for PSM.

Abbreviations: MI, multiple imputation; IPTW, inverse probability of treatment weighting; SMRW, standardized mortality ratio weighting; PA, pairwise algorithmic.

Table S.5 E-values for effectiveness of FZJD after excluding cases progressed to severe COVID-19 within 5 days in Non-FZJD users

| Analyses | E value **^a^** | Lower ^b^ |
| --- | --- | --- |
| Multivariable analysis | 4.312 | 1.530 |
| Multivariable analysis after MI | 3.973 | 3.973 |
| Propensity score analyses |  |  |
| Adjusted for propensity score | 7.464 | 2.082 |
| With matching | 7.799 | 1.564 |
| With IPTW | 6.868 | 1.530 |
| With SMRW | 4.484 | 1.250 |
| With PA | 8.560 | 1.702 |

**^a^** Results are E-values for the adjusted ORs in **Table S4**

^b^ Results are E-values for the upper bound of the CI of adjusted OR

Abbreviations: MI, multiple imputation; IPTW, inverse probability of treatment weighting; SMRW, standardized mortality ratio weighting; PA, pairwise algorithmic.

E-value ranged from 3.973 to 8.560, indicating that strong concurrent confounders are required to change the observed OR.
